# Microstructure and Corrosion Behavior of Iron Based Biocomposites Prepared by Laser Additive Manufacturing

**DOI:** 10.3390/mi13050712

**Published:** 2022-04-30

**Authors:** Yan Zhou, Lifeng Xu, Youwen Yang, Jingwen Wang, Dongsheng Wang, Lida Shen

**Affiliations:** 1 Key Laboratory of Construction Hydraulic Robots, Anhui Higher Education Institutes, Tongling University, Tongling 244061, China; zhouyan099@163.com (Y.Z.); abc402@163.com (L.X.); wangjingwener@126.com (J.W.); 2 Institute of Bioadditive Manufacturing, Jiangxi University of Science and Technology, Nanchang 330013, China; 3 Jiangsu Key Laboratory of Precision and Micro-Manufacturing Technology, Nanjing University of Aeronautics and Astronautics, Nanjing 210016, China; ldshen@nuaa.edu.cn

**Keywords:** iron bone implant, zinc sulfide, degradation properties, passivation film, laser powder bed fusion

## Abstract

Iron (Fe) has attracted great attention as bone repair material owing to its favorable biocompatibility and mechanical properties. However, it degrades too slowly since the corrosion product layer prohibits the contact between the Fe matrix and body fluid. In this work, zinc sulfide (ZnS) was introduced into Fe bone implant manufactured using laser additive manufacturing technique. The incorporated ZnS underwent a disproportionation reaction and formed S-containing species, which was able to change the film properties including the semiconductivity, doping concentration, and film dissolution. As a result, it promoted the collapse of the passive film and accelerated the degradation rate of Fe matrix. Immersion tests proved that the Fe matrix experienced severe pitting corrosion with heavy corrosion product. Besides, the in vitro cell testing showed that Fe/ZnS possessed acceptable cell viabilities. This work indicated that Fe/ZnS biocomposite acted as a promising candidate for bone repair material.

## 1. Introduction

Metal materials have excellent comprehensive mechanical properties (high strength, toughness, fatigue resistance) and good processing and forming ability [1,2]. Thus, medical metal implants have been widely used in the field of orthopedics. Degradable metals not only have excellent comprehensive mechanical properties, but also the degradation products can be absorbed by the human body [3,4]. As a new type of medical implant, it is able to be completely degraded and absorbed in the human body after service in vivo, avoiding the pain of patients’ secondary operation. Among the several representative degradable metal, iron (Fe) has gained intensive attention recently [5,6]. It is an essential nutrient element and participates in a variety of metabolic processes, which is able to maintain the normal function of bone cells. However, its degradation rate is too slow, which will hinder the growth of new bone as an implant.

Destroying the passive film is an effective way to accelerate its degradation of metallic matrix. Owing to the multivalent character of sulfur (S), sulfide is able to generate various S-containing species, which can attach on the metal surface and induce severe damaging effect on the passive film [7]. Previously, some scholars studied the S-induced corrosion of Fe-based metal material and indicated that the adsorbed S catalyzes the metal dissolution, thereby resulting in a decreased dissolution activation energy and accelerated anodic dissolution process [8]. It was also reported that the S-containing species changed the film properties including the semiconductivity, doping concentration, film dissolution rate, and film composition. Particularly, the synergistic effect of S species and chloride (Cl) was also confirmed by previous research [9].

Among S-containing species, zinc sulfide (ZnS) possessed relatively good biocompatibility and water solubility [10,11]. Basing on the above consideration, in this work, ZnS was incorporated into Fe implants aiming to accelerate the corrosion of Fe matrix. Meanwhile, Zn ion, as a trace element, could promote cell proliferation and differentiation, which was expected to improve the biocompatibility of Fe implant [12]. The Fe based implant was fabricated by the laser powder bed fusion (LPBF) technique. LPBF is a powder bed melting technology. The focused laser beam selectively melts the powder layer by layer to produce the required geometry. Since LPBF meets the requirements of high melting point, high dimensional accuracy, high performance and design flexibility, it has become the main additive manufacturing technology of metal implants [13,14,15]. The microstructure, corrosion behavior, and biocompatibility of Fe/ZnS composite fabricated by LPBF were investigated. Additionally, the corrosion mechanism was deeply studied.

## 2. Materials and Methods

### 2.1. Original Materials and Laser Powder Bed Fusion (LPBF) Process

Sphere Fe powder (mean particle size 35 μm) and ZnS powder (5–10 μm) were utilized in this work. Fe and ZnS (9 wt %) were mixed by a miniature planet ball mill (PULVERISETTE 6, Fritsch, Germany). The ball mill was operated at a rotation speed of 220 rpm for 2 h, with a 15 min pause every half an hour. During operation, high purity argon (99.9%) was offered to reduce the oxidation.

The mixed powder was adopted to fabricate Fe/ZnS biocomposite using LPBF system, which was consisted of a fiber laser and a computer control system. A series of pilot experiments were carried out before the LPBF experiments to obtain an optimized processing parameter and as follows: laser power 210 W, scanning rate 80 mm/s, hatching space 50 μm and layer thickness 50 μm.

### 2.2. Microstructural Characterization

The LPBF-processed parts were grounded and polished using SiC paper. The microstructure was characterized using a scanning electron microscopy (SEM, Zeiss, Oberkochen, Germany) equipped with an energy dispersive spectroscopy (EDS). The phase composition was determined using X-ray diffractometer (XRD, D8 Advance, Bremen, Germany) with Cu Kα radiation at 45 kV and 40 mA. The scanning range was 20–90°, and the scan rate was 8°/min.

### 2.3. Electrochemical Tests

An electrochemical experiment was carried out to study the corrosion behavior. The self-prepared simulated body fluid (SBF) was used at testing solution. A three-electrode system was adopted in electrochemical tests. The nominal chemical composition of SBF was listed in Table 1. The system consisted of platinum as counter electrode, saturated calomel as reference electrode and the test part as working electrode. The initial open-loop circuit (OCP) tests were firstly performed. Then, the Tafel polarization curve was recorded at a rate of 0.05 mV/s. The corrosion rate (*Pi*) was determined by corrosion current (*I_corr_*):

*Pi* = 3.27 × 10^−3^ × *I_corr_* E/ρ (1)

E was the weight equivalent, and ρ was the material densigty. Besides, the electrochemical impedance spectroscopy (EIS) testing was carried out within the scope of 0.01 Hz to 1000 kHz. Zsimpwin software was adopted to analyze the result. Furthermore, the transient time-current curve was determined at 1 mV/s. The Mott-Schottky curve was recorded to study the semiconductor properties of the corrosion film.

### 2.4. Immersion Tests

SBF immersion testing was performed to further study the degradation behavior of as-built parts. The parts were immersed in SBF at an exposure ratio of 0.1 cm^2^/mL. After immersion for 7, 14, and 28 days, the parts were washed with distilled water and then observed by SEM. The samples were washed using 200 g/L of CrO_3_ solution to remove corrosion products. Subsequently, the surface morphology was investigated by an atomic force microscope (AFM, Veeco Instruments, Plainview, NY, USA). Meanwhile, the corrosion rate (*Cr*) was calculated by using the weight loss method after immersion tests.

### 2.5. Cytotoxicity Evaluation

MG-63 cells were used to evaluate the cytotoxicity of Fe-based biocomposite. Dulbecco’s modified Eagle’s medium (DMEM) containing 10% fetal bovine serum, 100 units/mL penicillin and 100 mg/mL streptomycin was used as culture medium. The as-built samples were sterilized, and then immersed in DMEM for three days to obtain the extracts. Then, the cells were incubated in a 96-well plate for 1 day using DMEM, subsequently substituted by the 100 centration extracts. After one, four, and seven days, Calcian-AM reagent was used to stain the cells for 15 min. Afterwards, the cells were captured using a fluorescence microscopy (BX60, Olympus, Tokyo, Japan). Furthermore, the cell counting kit-8 (CCK-8) reagent was added into the culture medium and continued to incubate for 3 h. Finally, the absorbance was detected by a microplate reader at 450 nm. 

### 2.6. Statistical Analysis

In this work, the immersion tests, electrochemical experiments and cell experiments were performed three times. The data was expressed as means ± errors. The significant difference was investigated suing SPSS soft, in which *p* less than 0.05 was determined to be of significant difference.

## 3. Results

### 3.1. Microstructural Feature of LPBF-Processed Parts

**Figure 1:**
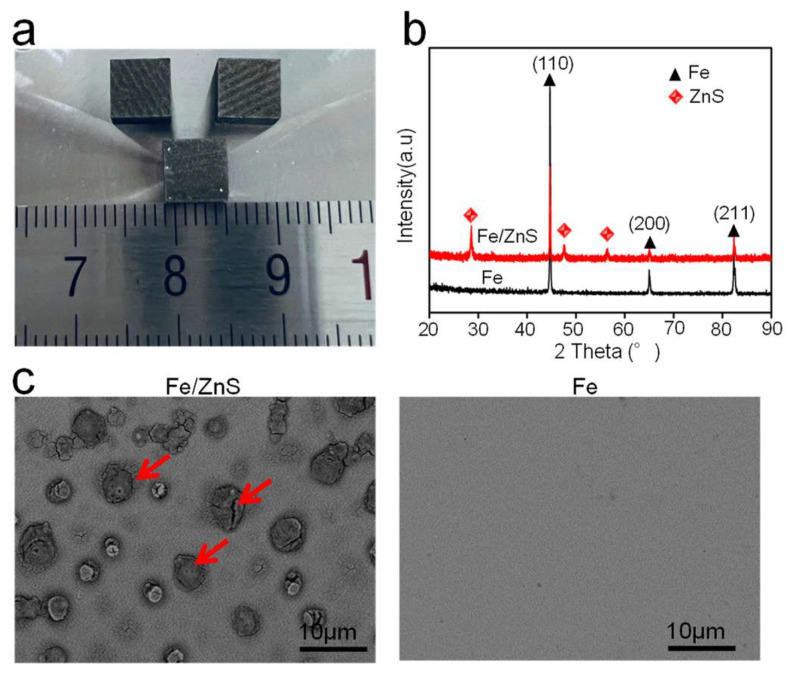
(**a**) LPBF processed Fe-based parts; (**b**) the XRD spectrum and (**c**) SEM for Fe and Fe/ZnS biocomposite showing the microstructure. The ZnS particles were marked by the red arrows.

The LPBF-processed bulk parts were shown in Figure 1a, and the corresponding XRD spectrum was depicted in Figure 1b. Results showed that strong peaks corresponding to α-Fe phase was observed for Fe and Fe/ZnS parts. Besides, some strong peaks corresponding to ZnS phase presented in Fe/ZnS composite. The microstructure was observed by SEM, as shown in Figure 1c. No obvious holes and cracks were observed in the matrix of as-built parts, indicating their good forming quality. For the LPBF of the metal parts, the relatively high porosity is easily generated due to the insufficient liquid phase or severe molten pool evaporation, thereby reducing the performance including mechanical properties and corrosion resistance [16]. However, our SEM analysis showed the high densification rate was obtained. It was reported that the stable molten pool behavior could be achieved under the optimized laser parameters, so as to promote the densification of the parts [17]. For the Fe/ZnS biocomposite, the ZnS particles (as marked by the red arrows) were uniformly distributed in the matrix.

### 3.2. Degradation Behavior

**Figure 2:**
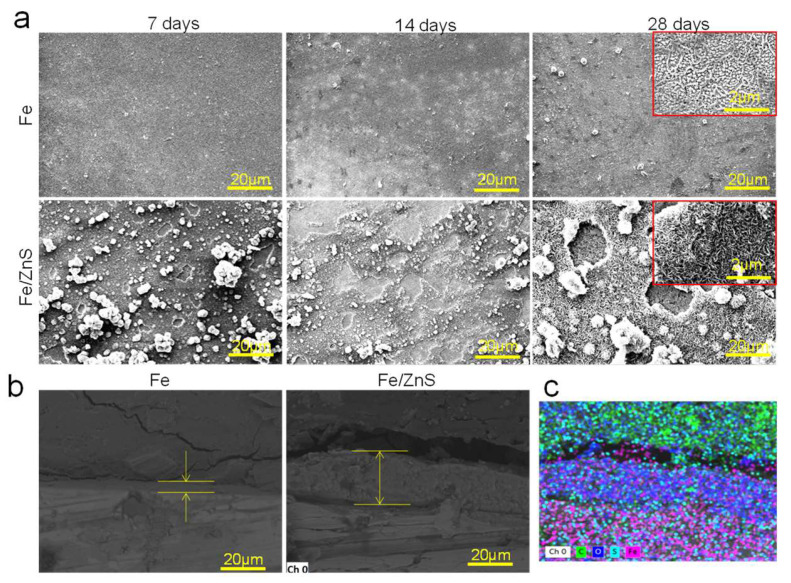
(**a**) SEM showing the typical corrosion surface of Fe and Fe/ZnS composite after immersion in SBF; (**b**) the cross section of corrosion production film and (**c**) the corresponding EDS mapping.

The corroded surface after immersion for 7, 14, and 28 days have bee shown in Figure 2a. Flat corrosion surface with little degradation product was observed for Fe part over the whole immersion period. As a comparison, a large amount of corrosion product was presented on the Fe/ZnS biocomposite, and a porous film with numerous corrosion pits was also observed. The corrosion pits obviously became deepened and expanded at day 28, accompanied by partial products falling off. The cross section after immersion for 28 days was examined by SEM, as exhibited in Figure 2b. Clearly, the thin corrosion film with a thickness of only ~4.8 μm was observed for Fe part. As for Fe/ZnS biocomposite, the thickness of corrosion film increased to ~23.8 μm. The element mapping analysis showed that the corrosion film mainly contained Fe and O elements, as shown in Figure 2c. Previous studies reported that the corrosion products on Fe matrix mainly contained oxides and hydroxides of Fe [18].

**Figure 3:**
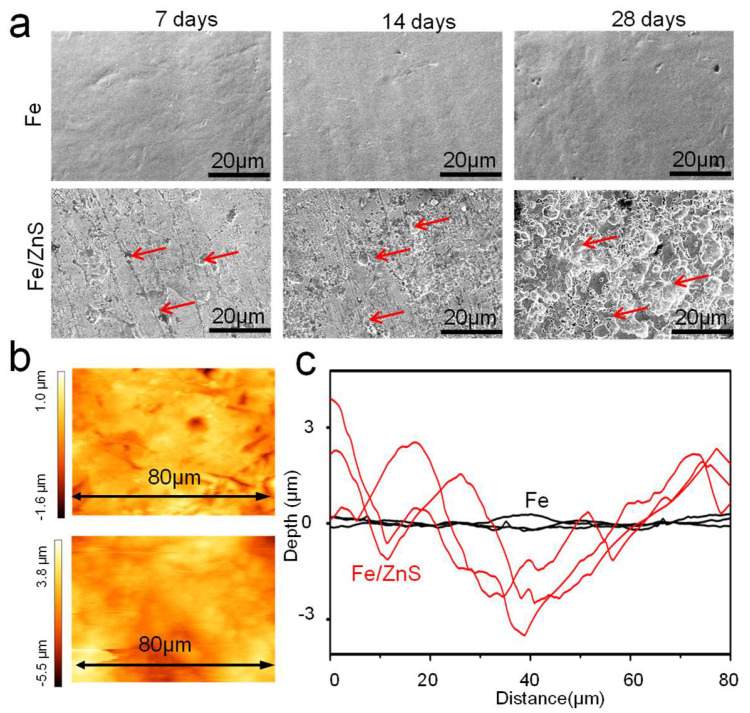
(**a**) The surface topography after removing corrosion products for Fe and Fe/ZnS composite, (**b**) AFM images and (**c**) the surface roughness profiles. The corrosion pits were marked by the red arrows.

The corrosion surface after removing the corrosion product was also observed by SEM, as shown in Figure 3a. It could be seen that the corroded surface of Fe part showed small change after immersion in SBF for 7, 14, and 28 days. As a comparison, massive corrosion pits, as marked by the arrows, appeared on the matrix surface of Fe/ZnS part with the extension of immersion time. Clearly, the pits dimension gradually increased to 5–10 μm. The surface roughness after immersion for 28 days was shown in Figure 3b. For Fe part, the gradient range of surface roughness was between −1.6 and 1.0 μm. As for Fe/ZnS biocomposite, the gradient range was extended to −5.5~3.8 μm. Besides, the surface roughness profiles showed that the curve of Fe/ZnS biocomposite fluctuated sharply as compared with that of Fe, as shown in Figure 3c. It was indicated that the matrix of Fe/ZnS was severely corroded. According to the mass loss during immersion for 28 days, the degradation rates of Fe and Fe/ZnS were calculated to be ∼0.05 and 0.14 mg/cm^2^/year, respectively (Table 2). The significance analysis showed that the corrosion rates of Fe/ZnS was significantly higher than that of Fe (*p* < 0.05).

### 3.3. Electrochemical Behavior

**Figure 4:**
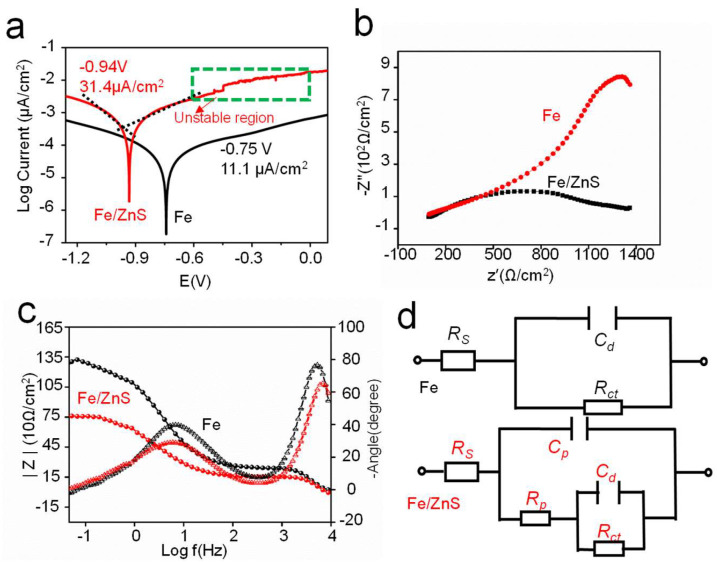
(**a**) The potentiodynamic polarization curves, (**b**) EIS spectra, (**c**) Bode plots and (**d**) the equivalent circuits obtained from electrochemical tests.

The degradation mechanism of Fe/ZnS and Fe was investigated by electrochemical tests. The obtained polarization curves were shown in Figure 4a. The corrosion potential (Ecorr) and corrosion current density (Icorr) were also calculated by Tafel extrapolation method, and the result was shown in Figure 4a. The Ecorr value of Fe and Fe/ZnS composite were −0.75 V and −0.94 V, respectively. And the Icorr value of Fe/ZnS composite was significantly enhanced to 31.4 ± 0.9 μA/cm^2^ as compared with that of Fe. The corrosion rate of Fe and Fe/ZnS calculated by the electrochemical parameters were ∼0.25 and 0.72 mm/year, respectively, as shown in Table 2. Particularly, for Fe/ZnS composite, there was a typical pitting area in the region of anode polarization curve, as marked in Figure 4a. It was suggested that the addition of ZnS was effectively pierced through the dense corrosion layer, and changed the corrosion type from surface corrosion to pitting corrosion. The electrochemical impedance spectra are shown in Figure 4b. The Fe/ZnS composite showed small impedance value than that of Fe, which also verified its low corrosion resistance. For the Fe part, there was only one impedance loop in the whole frequency range, indicating the formation of compact oxide film during corrosion. However, Fe/ZnS composite had a relatively small capacitive arc and impedance moduli at low frequency region, reflecting a low electron transfer resistance. Furthermore, the phase angle value and impedance value in the Bode plots were further applied to indicate the stability of corrosion films, presented in Figure 4c. For Fe/ZnS composite, the phase angle value and impedance value were smaller, which indicated the corrosion film was a loose membrane structure. It was believed that the passive film was continuously self-destroyed, thus reducing the protection efficiency for Fe/ZnS composite. The equivalent circuits of electrochemical tests for Fe and Fe/ZnS were obtained, shown in Figure 4d. There was only a semicircle in the EIS diagram, which meant only exist a corrosion layer on the Fe matrix. There were two semicircles for the Fe/ZnS composite, which indicated another transfer reaction except. Generally, it was remarked by a double-layer capacitance *C*_d_ and charge transfer resistance *R*_ct_. *R*_ct_, and *C*_d_ were the resistance and capacitance of the passive film, respectively. As for the Fe/ZnS composite, a relatively low *R*_ct_ and *C*_d_ revealed its high charge transfer ability of the product layer.

**Figure 5:**
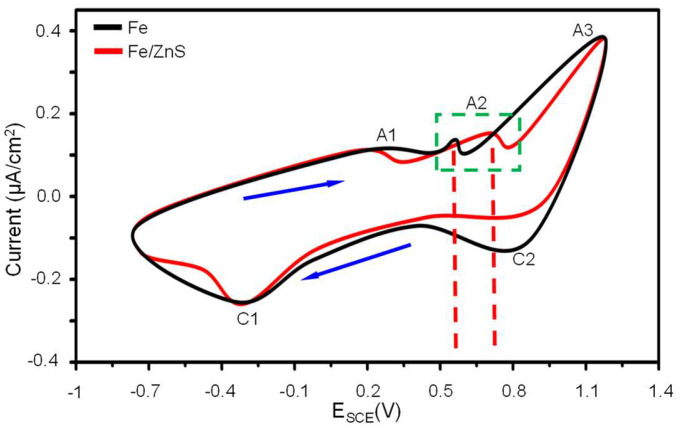
The cyclic voltammetry curves obtained from electrochemical tests.

The cyclic voltammetry (CV) curves of Fe and Fe/ZnS samples were presented in Figure 5. The A curve was the anodic branch while C curve was the cathodic branch. Three anodic current peaks of A1, A2, and A3 were observed during the anodic scan process. The A1 peak was considered to correspond to the electro-oxidation of Fe to Fe^2+^, which was represented the initiation of passivity formation. There was no significant difference between Fe and Fe/ZnS samples at the A1 peak. The A2 peak was characterized with the oxidation process from Fe^2+^ to Fe^3+^, which was represented the formation process of a denser Fe_2_O_3_ passivation film. The A3 peak involved the transfer reaction and oxygen evolution reaction on behalf of the dissolution of Fe anode. At the A2 peak, the potential of Fe was around 0.6 V, but for Fe/ZnS samples the potential shifted to 0.7 V and caused a faster anodic reaction. With the positive shift of the potential, the anodic reaction was activated, the corrosion current increased gradually. Generally, the potential was more positive (which meant a higher anode activation energy), and thus the oxidation reaction was easier to carry out. Therefore, it was indicated that S destroyed the formation of the passivation layer and caused the continuous exposure of Fe matrix to corrosion solution, thus accelerating degradation.

### 3.4. In Vitro Cytotoxicity

**Figure 6:**
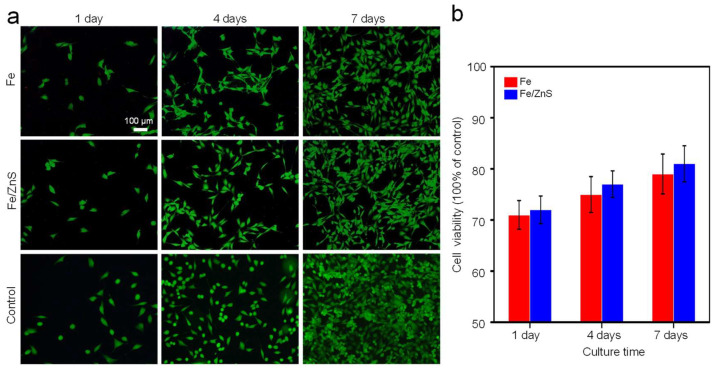
(**a**) The cell fluorescent images and (**b**) cell viability obtained by CCK-8 testing (n.s.).

As exhibited in Figure 6a, very few dead cells were observed during the whole incubation period. With the culture time extending, the cells were gradually increased, which proved their normal development. After seven days’ culture, most the cells presented representative fusiform shape, which was known as a healthy morphology. The cell viability was quantitatively studied, as shown in Figure 6b. At day, the cell viability was relatively low, since the cells could not adapt to the new environment with high concentration of metal ions. However, with the increase of culture time, the cell activity gradually increased to 80%, which confirmed that it had acceptable biocompatibility. Moreover, there was no significant difference between the two groups.

## 4. Discussion

Bone implants should have a through-hole structure similar to natural bone, so as to provide a necessary microenvironment for cell growth, angiogenesis, and new bone growth after implantation [19,20]. It is generally believed that the pore size suitable for bone tissue growth ranges from 200 to 600 μm. At the same time, the porosity should reach more than 70%, which is conducive to cell adhesion, extracellular matrix deposition, oxygen, nutrition entry, and metabolite discharge [21,22]. In addition, bone implants should also have personalized and accurate shapes to improve the adaptability of implants in the process of operation and the effect of postoperative treatment [23,24].

**Figure 7:**
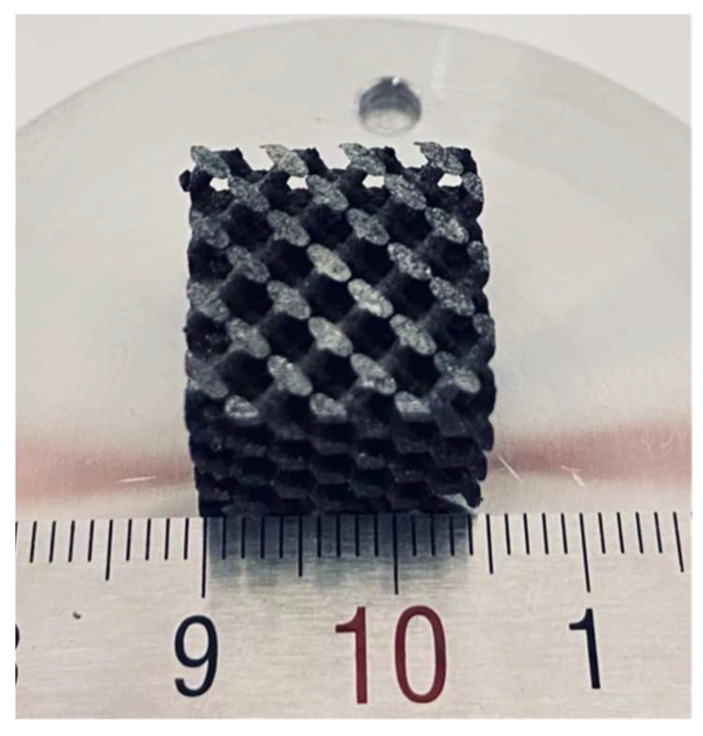
Laser additively manufactured Fe/ZnS composite scaffold with porous structure.

LPBF as a typical additive manufacturing technology is especially suitable for the high-precision and high-efficiency manufacturing of personalized porous implants [25,26]. Specifically, we can use digital medical technology to conduct three-dimensional scanning of the bone defect, and then design the bone defect model through computer-aided technology. Finally, we can manufacture customized porous implants through LPBF technology, as shown in Figure 7. In light of this, the additive manufacturing technology represented by LPBF has carried out an upsurge of applied research in medical fields such as orthopedics, dentistry, and cardiovascular stents [27,28,29].

**Figure 8:**
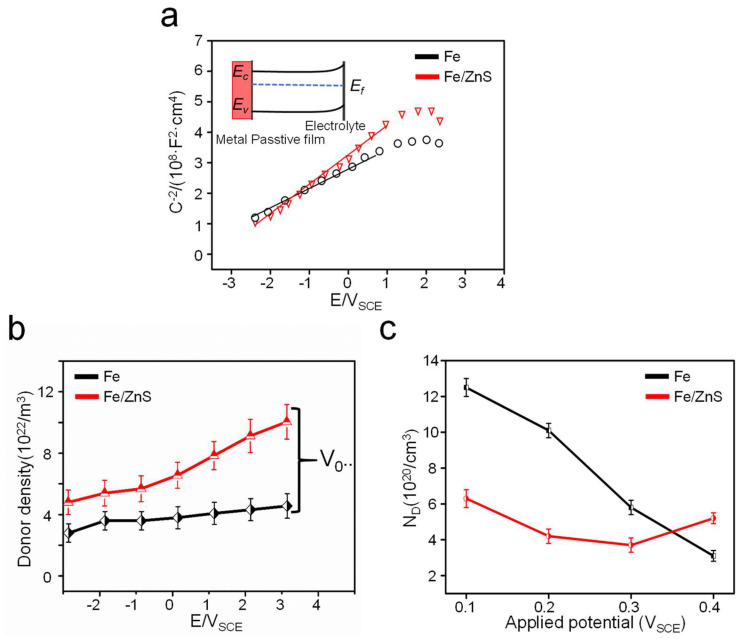
(**a**) Mott-Schottky results of Fe and Fe/ZnS composite in SBF solution, (**b**) the oxygen vacancy density variation of passive film, and (**c**) the calculated defects density of the passive film.

On the other hand, bone implants also demand suitable degradation rate to match the growth rate of new bone tissue [30]. In the present study, ZnS was introduced into the Fe matrix to accelerate degradation. Both the immersion tests and electrochemical tests proved that the Fe/ZnS showed an enhanced degradation rate, since the ZnS promoted the collapse of the passive film. In fact, the collapse of the passive film was closely related with its electronic properties. Herein, the typical Mott-Schottky plots were used to characterize the electronic property of the passive film, as shown in Figure 8a. The positive slopes of the two curves indicated that the passive film exhibited n-type semiconductor behavior. It was attributed to the present of minority carriers in the corrosion layer, and its defects were composed of oxygen vacancies. The variation range of passive film defect density with polarization potential has been shown in Figure 8b. It was obvious that the donor density in the corrosion layer increased with the increase of polarization voltage, thus resulting in the increase of vacancy accumulation between the Fe matrix and passive film interface. Therefore, the corrosion pits were formed in the Fe matrix, as verified by the corrosion surface and electrochemistry experiments. The calculated oxygen vacancy density of the passive film also demonstrated these results, as shown in Figure 8c. The electronic conductivity of the general corrosion product film was related with the defect concentration, according to other research result. The defect concentration for Fe/ZnS composite could enhance the electron transmission of the passive film, thereby resulting in the formation of corrosion pits on the surface. In this condition, the protective effect of the passive film was gradually weakened, which further accelerated the damage of passive film and the corrosion of the Fe matrix.

In the present work, the incorporation of ZnS changed the properties of the passive film. It was believed that the added ZnS would undergo a disproportionation reaction in SBF, and formed S-containing species such as S^2−^, HS^−^, and S_2_O_3_^2−^ ions [31]. The catalysis of S^2−^ absorbed on Fe matrix through the anodic reaction led to an increase in anodic dissolution kinetics, resulting in a higher maximum current density. With a positive shift of the anode potential, S-containing species oxidized from low to high valence, resulting in the formation of adsorbed S elemental and thiosulfate ion. Previous studies demonstrated that S-containing species had a detrimental effect on Fe matrix. Marcus et al. suggest that the adsorbed S weakened the metal-metal bond, resulting in lower activation energy for the dissolution of surface metal atoms [32]. In addition, the adsorbed S might hinder or delayed passivation, as it hindered the available sites for hydroxyl ion adsorption, a precursor for passive film formation [7]. Furthermore, the formation of S-containing phases led to localized acidification, which also contributed to the degradation of the Fe matrix [33]. As our electrochemical tests proved, Fe/ZnS showed a significantly enhanced corrosion current density, which was almost five times that of the Fe part.

Generally, bone implants not only require a porous structure and appropriate degradation rate but also need good biocompatibility [34,35]. Both Fe and Zn were the trace elements of human body, and possessed good biocompatibility. As our cell testing proved, the cell viabilities of Fe/ZnS of Fe extracts were higher than 80% at day seven, which was acceptable as bone implant. It was worth noting that Fe/ZnS had a higher degradation rate, that was, a higher ion concentration for Fe/ZnS group. However, the cellular activity for Fe/ZnS group was still higher than that of the Fe group. This may be due to the fact that the released Zn ions exerted a positive role. Zn plays a significant role in the formation, development, mineralization, and maintenance of healthy bones [36,37]. Thus, it was expected that Zn ions released from the Fe/ZnS composites could promote cell growth and proliferation.

## 5. Conclusions

In the present work, ZnS was incorporated into Fe based implants to improve their degradation behavior. An Fe based biocomposite was fabricated by LPBF. The Mott-Schottky test analysis indicated that S destroyed the formation of the passivation layer and caused the continuous exposure of Fe matrix to corrosion solution, thus accelerating the degradation rate. After immersion in SBF for 28 days, heavy corrosion product and porous film with numerous corrosion pits presented on the Fe/ZnS composite, which revealed that it undergone severe corrosion. Besides, the incorporated ZnS had no significant effect on the biocompatibility for Fe based implants. All of the results showed that the Fe/ZnS biocomposite was a good choice for use as a bone repair material.

## Figures and Tables

**Table 1:** Chemical composition of SBF.

Composition	NaCl	NaHCO_3_	KCl	K_2_HPO_4_·3H_2_O	MgCl_2_·6H_2_O	CaCl_2_
Weight (g/L)	8.035	0.355	0.225	0.231	0.311	0.292

**Table 2:** The corrosion rates calculated from immersion and electrochemical tests.

Samples	*Cr* (mg/cm^2^/year)	*Pi* (mm/year)
Fe	0.05 ± 0.01	0.25 ± 0.02
Fe/ZnS	0.14 ± 0.03	0.72 ± 0.05
